# Comparative Secretome Analysis of Mesenchymal Stem Cells From Dental Apical Papilla and Bone Marrow During Early Odonto/Osteogenic Differentiation: Potential Role of Transforming Growth Factor-β2

**DOI:** 10.3389/fphys.2020.00041

**Published:** 2020-03-06

**Authors:** Shi Yu, Jingzhi Li, Yuming Zhao, Xiaoxia Li, Lihong Ge

**Affiliations:** Department of Pediatric Dentistry, Peking University School and Hospital of Stomatology, Beijing, China

**Keywords:** transforming growth factor-β2 (TGFβ2), secretory proteins, odonto/osteogenic differentiation, stem cells from dental apical papilla (SCAPs), bone marrow–derived stem cells (BMSCs)

## Abstract

To understand the functions of secretory proteins in odontogenesis and to further the understanding of the different molecular events during odontogenesis and osteogenesis, we induced the odonto/osteogenic differentiation of stem cells from dental apical papilla (SCAPs) and bone marrow–derived stem cells (BMSCs) *in vitro* and compared the expression of secretory proteins during early odonto/osteogenic differentiation using high-performance liquid chromatography with tandem mass spectrometry. The results revealed significant changes by at least 50% in 139 SCAP proteins and 203 BMSC proteins during differentiation. Of these, 92 were significantly upregulated and 47 were significantly downregulated during the differentiation of SCAPs. Most of these proteins showed the same trend during the differentiation of BMSCs. Among the proteins that showed significantly changes during the differentiation of SCAPs and BMSCs, we found that transforming growth factor-β2 (TGFβ2) is a key protein in the network with powerful mediation ability. TGFβ2 was secreted more by SCAPs than BMSCs, was significantly upregulated during the differentiation of SCAPs and was significantly downregulated during the differentiation of BMSCs. Furthermore, the effects of recombinant human TGFβ2 and TGFβ1 on the odonto/osteogenic differentiation of SCAPs and BMSCs were investigated. Real-time reverse transcription polymerase chain reaction (RT-PCR) and western blotting data revealed that TGFβ2 enhanced the odontogenic-related markers [dentin sialophosphoprotein (DSPP), dentin matrix protein 1 (DMP1)] and inhibited the osteogenic-related marker bone sialoprotein (BSP) in SCAPs, whereas TGFβ1 enhanced the BSP expression and inhibited the DSPP and DMP1 expression at early odonto/osteogenic differentiation of SCAPs. However, in BMSCs, TGFβ2 enhanced the expression of alkaline phosphatase (ALP), runt-related transcription factor 2 (RUNX2), DSPP, and DMP1, whereas TGFβ1 enhanced the expression of ALP and RUNX2, with no significant intergroup difference of DSPP at the early odonto/osteogenic differentiation of BMSCs. TGFβ2 is a potentially important molecule with a distinct function in the regulation of odontogenesis and osteogenesis.

## Introduction

The treatment of immature permanent teeth with necrotic pulp is a challenging task for dental professionals. The young pulpless tooth with an open apex frequently has a thin and fragile wall, which increases the risk of tooth fracture and makes it difficult to obtain the necessary apical seal using conventional treatment methods. A novel treatment of pulp revascularization/regeneration is an effective approach for inducing root maturation (Iwaya et al., 2001; Banchs and Trope, 2004). During revascularization/regeneration procedures, the evoked-bleeding step leads to a substantial influx of stem cells from dental apical papilla (SCAPs) into the root canal, which contributes to root development and apical closure (Cao et al., 2015). However, several histological findings reported that most tissues formed in the canal of the revitalized immature permanent tooth lack organized pulp–dentin complex and consist of ectopic bone, fibrous tissues, and cementum (Martin et al., 2012; Saoud et al., 2015; Peng et al., 2017). In some cases, the root canal space became partly obliterated with ectopic bone (Martin et al., 2012; Saoud et al., 2015). It is presumed that stem cells from alveolar bone would migrate into the root canal and initiate ectopic calcification deposition (Cao et al., 2015; Saoud et al., 2015). Mesenchymal stem cells (MSCs) from different dentoalveolar tissues are unique and retain identities from their direct tissue sources. An understanding of the molecular mechanisms underlying the differentiation of odontoblasts and osteoblasts and their secretion of a mineralized matrix is fundamental for developing strategies for the regeneration of hard tissue. It is necessary to find some important factors to regulate the differentiation of stem cells into odontoblasts and osteoblasts.

SCAPs are considered to be the source of primary odontoblasts (Huang et al., 2008; Sonoyama et al., 2008). The interruption of SCAP development results in halted root development (Chueh and Huang, 2006). Stem cell–based approaches and tissue engineering have demonstrated that SCAPs can differentiate into odontoblasts and regenerate a continuous layer of dentin with a uniform thickness on an existing dentinal wall *in vivo* (Sonoyama et al., 2008; Huang et al., 2010). During bone formation, osteoblasts are derived from bone marrow–derived stem cells (BMSCs). BMSCs can differentiate into osteoblasts after culture in mineral-inducing medium (Dj, 1997; Bianco et al., 2001). When BMSCs are transplanted into the subcutaneous space of immunocompromised mice, they form bone-like complexes, while if the seeding cells are replaced by dental pulp stem cells, they form dentin-like complexes (Gronthos et al., 2000).

Bone and dentin are mineralized tissues mainly composed of calcium hydroxyapatite and type I collage. The process of hydroxyapatite nucleation and collagen mineralization is controlled by proteins that are categorized as non-collagenous proteins (N) (Young et al., 1992). Dentin matrix proteins are a group of NCPs found in the extracellular matrix (ECM) of dentin and bone albeit in different quantities. Dentin phosphophoryn (DPP) and dentin sialoprotein (DSP) are highly specific dentin matrix proteins. The amount of DSP in bone is about 1/400 of that in dentin (Qin et al., 2002). DSP and DPP are proteins encoded by the gene dentin sialophosphoprotein (DSPP). DSPP defects cause dentinogenesis imperfecta and are not associated with bone abnormalities (Zhang et al., 2001; Kim et al., 2005). Dentin matrix protein 1 (DMP1) is one of the early genes expressed during the commitment of neural crest–derived cells into odontoblasts. It is showed that DMP1 is able to bind specifically with the DSPP promoter and activate its transcription (Narayanan et al., 2006). Previous studies have suggested that stem cell–mediated bone and dentin regeneration are regulated by distinct mechanisms, although the differences remain unclear (Batouli et al., 2003). The process by which MSCs differentiate into osteoblasts is regulated by coordinated extracellular signals from autocrine and paracrine loops (Zaidi, 2007; Kim et al., 2013). The secreted ECM provides a series of signals to cells, regulating all aspects of their phenotype from morphology to differentiation.

The MSCs derived from different tissues have distinct characteristics. *In vitro*, MSCs retain the capacity to regenerate unique microenvironments similar to those from which they are derived (Gronthos et al., 2000). Key lineage decisions are made during the early stages of mesenchymal differentiation. Given that the initial event in osteogenesis is the determination of MSCs to become osteoprogenitor cells, we profiled the secretomes of SCAPs and BMSCs after inducing their odonto/osteogenic differentiation *in vitro* and compared the expression of the secreted proteins using isobaric chemical tags (tandem mass tags, TMTs) and high-performance liquid chromatography with tandem mass spectrometry (HPLC-MS/MS).

## Materials and Methods

### Sample Collection and Cell Culture

Normal human impacted third molars with immature roots and normal human bone marrow obtained from the mandibular alveolar bone (*n* = 5) were collected from healthy patients (aged 16–30 years). Sample collection was approved by the Ethics Committee of the Health Science Center of Peking University (Beijing, China; IRB00001052-11060 and PKUSSIRB-201734036). SCAPs were isolated from dental apical papilla tissue and BMSCs from bone marrow. Cultures of MSCs were maintained in a-modified Eagle’s minimum essential medium (aMEM; Gibco, United States) supplemented with 10% fetal bovine serum (FBS; Gibco) in 5% CO_2_ at 37°C. Three to five passages of cells were used in our experiments. SCAPs and BMSCs were characterized by flow cytometry with antibodies for CD73, CD90, CD105, CD146, and CD34 (BD Biosciences, United States) as previously described (Yu et al., 2016).

MSCs were grown to confluence prior to the induction of odonto/osteogenesis. We used a mineral-inducing medium composed of aMEM supplemented with 10% FBS, 10 mM β-glycerophosphate (Sigma-Aldrich, United States), 50 mM ascorbate-2-phosphate (Sigma-Aldrich, United States), and 100 nM dexamethasone (Sigma-Aldrich, United States). To assess mineralization, MSCs maintained in this medium for 0, 6, 9, and 14 days were fixed with 70% ethanol and stained with 2% alizarin red (Sigma-Aldrich, United States).

### Preparation of Conditioned Media and Protein Digestion

SCAPs and BMSCs were seeded on 100 mm plates at 20,000 cells/cm^2^. When they reached 90% confluence, the medium was changed to growth medium or mineral-inducing medium. Six days later, the cells were washed five times with phosphate-buffered saline and cultured in serum-free medium for 24 h. The conditioned media were collected after centrifugation at 1,000 rpm for 10 min to remove the cellular debris and passed through a 0.22 μm filter. The samples were concentrated, air-dried, re-dissolved in triethylammonium bicarbonate (Promega, United States), and reduced with dithiothreitol at 55°C for 1 h. Next, iodoacetamide (Promega) was added to the samples, which were maintained for 1 h at room temperature in the dark. The protein concentration was determined using bicinchoninic acid (Thermo Fisher Scientific, United States) assay.

### TMT, HPLC-MS/MS Analysis, Database Search, and Quantitative Data Analysis

Samples were digested with sequence-grade modified trypsin (Promega) and labeled using a TMT reagent kit (Thermo Fisher Scientific, United States) (Thompson et al., 2003). Ten fractions were collected by high-pH separation using an Acquity UPLC system (Waters Corporation, United States) (Gilar et al., 2005). HPLC-MS/MS analysis, database search, and quantitative data analysis were performed as previously described (Yu et al., 2016). A peptide identification was accepted if the false discovery rate (FDR) was < 1.0%, and a protein identification was accepted if the probability was >66.0% and it contained at least one identified peptide. Acquired intensities in the experiment were globally normalized across all acquisition runs. Individual quantitative samples were normalized within each acquisition run. The intensity of each identified peptide was normalized within the assigned protein. The reference channels were normalized to produce a 1:1 ratio. All normalization calculations were performed using medians to multiplicatively normalize the data. Differentially expressed proteins were determined using the Kruskal–Wallis test. String software^1^ was used to visualize protein interaction networks between the upregulated ECM proteins in SCAPs after induction. For functional annotation analysis, we used DAVID (Database for Annotation, Visualization, and Integrated Discovery), Bioinformatics Resources 6.7^2^, and the PANTHER (Protein Analysis Through Evolutionary Relationships) classification system^3^.

### Western Blotting Analysis

Thirty micrograms of protein was separated by 10% sodium dodecyl sulfate–polyacrylamide gel electrophoresis and transferred onto a polyvinylidene fluoride membrane, which was blocked with 5% (w/v) non-fat dried milk, incubated overnight at 4°C with primary antibodies (transforming growth factor-β2, TGFβ2, Abcam, United Kingdom; insulin-like growth factor 2, IGF2, Abcam; periostin, Abcam; alpha-1-antichymotrypsin, SERPINA3, Abcam; alkaline phosphatase, ALP, Servicebio, China; runt-related transcription factor 2, RUNX2, Abcam; DSPP, Santa, United Kingdom; DMP1, Bioss, United Kingdom), and reacted with horseradish peroxidase–conjugated secondary antibodies (Origene, China). Immunoreactive bands were visualized by enhanced chemiluminescence (Cwbiotech, China) at room temperature and digitized using the Fusion FX image analyzer (Viber Loumat, Germany).

### Real-Time Quantitative Reverse Transcription Polymerase Chain Reaction Analysis

Total RNA was extracted from cells using TRIzol reagent (Invitrogen, United States) according to the manufacturer’s instructions. cDNA was synthesized from oligo (dT) primers using a reverse transcriptase kit (Promega). The primer sequences were designed by a primer bank (Supplementary Table 1). Reverse transcription polymerase chain reaction (RT-PCR) was performed in triplicate in 96-well plates using a 7900HT Fast Real-Time system (Applied Biosystems, United States). The comparative cycle threshold (2^–ΔΔCT^) method was used to calculate the relative expression levels of the target genes.

### Statistical Analysis

Statistical analysis was performed using SPSS version 15.0 (SPSS, United States). Student’s *t*-tests were used to assess the significance of differences between groups. A *p* < 0.05 was considered significant. All experiments were repeated at least three times (*n* ≥ 3).

## Results

### Odontogenic/Osteogenic Differentiation of MSCs

**FIGURE 1:**
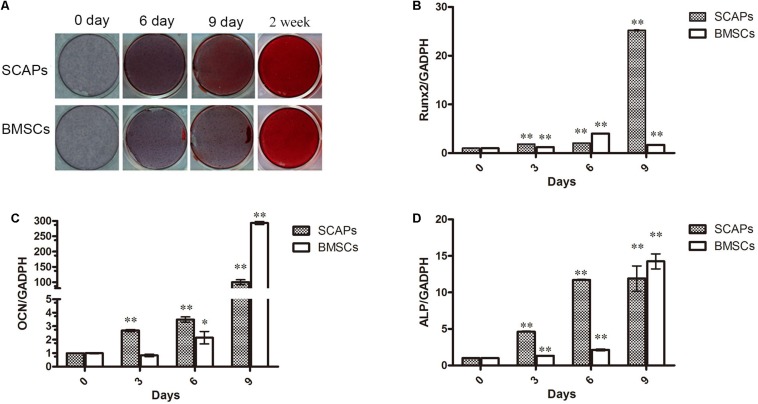
Stem cells from dental apical papilla (SCAPs) and bone marrow–derived stem cell (BMSCs) exhibit odontogenic/osteogenic differentiation. **(A)** Photographs of alizarin red–stained mineral deposits in the cultures of SCAPs and BMSCs after 6, 9, and 14 days in mineral-inducing medium. Mineral accumulation was observed from day 6 after induction. **(B–D)** Expression of odontogenesis/osteogenesis marker genes. The expression of osteocalcin (OCN), runt-related transcription factor 2 (RUNX2), and alkaline phosphatase (ALP) were increased after induction. PCR expression level is the fold change relative to the levels on day 0; all data are presented as the mean ± SD; ^∗^*p* < 0.05, ^∗∗^*p* < 0.01 versus day 0; each experiment was repeated three times.

Following culture of SCAPs and BMSCs in mineral-inducing medium, mineral accumulation was observed from day 6 after induction, as confirmed by alizarin red staining (Figure 1A). In addition, the expression of the odontogenesis/osteogenesis marker genes osteocalcin (OCN), RUNX2, and ALP were increased after induction (Figures 1B–D).

### The Secretome During the Early Odonto/Osteogenic Differentiation of MSCs

**FIGURE 2:**
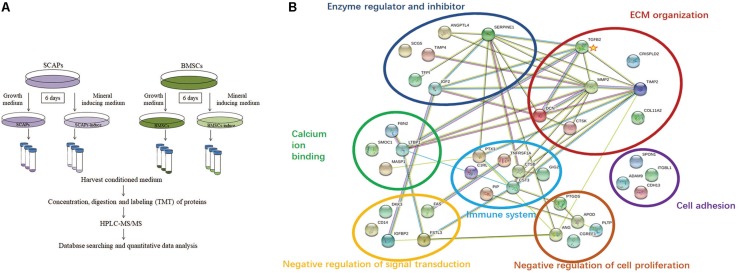
Bioinformatics analysis of the secretome. **(A)** Procedure workflow. **(B)** Functional network of upregulated extracellular proteins during odonto/osteogenic differentiation of SCAPs by String software. The proteins belonged to seven major functional classes. The interaction network of differentially expressed genes indicated that matrix metalloproteinase2, serpin family E member 1, metallopeptidase inhibitor 2, decorin, cystatin C, transforming growth factor-β2 (TGFβ2), and insulin-like growth factor 2 (IGF2) were the most important genes in the network with powerful mediation ability. Among these genes, TGFβ2 was a key node with interaction with six proteins.

To explore the secretory proteins during their early odonto/osteogenic differentiation, the conditioned media from SCAP, BMSC, SCAP-inducing, and BMSC-inducing groups were analyzed by HPLC-MS/MS (Figure 2A). A total of 2,046 proteins were detected in the conditioned media, with an FDR of <1.0%. Of the total, 1,172 were detected in all three experiments. These proteins and their identification parameters are listed in Supplementary Tables 2, 3.

In 139 of the identified proteins, we found significant differences of at least 50% (*p* < 0.05) during the odonto/osteogenic differentiation of SCAPs as well as in 203 proteins during the differentiation of BMSCs. Of the 139 proteins, 92 were significantly upregulated, and most also increased during the differentiation of BMSCs. Some of these are listed in Table 1. On the other hand, TGFβ2 and tumor necrosis factor receptor superfamily member 1A were significantly upregulated during the differentiation of SCAPs and significantly downregulated during the differentiation of BMSCs. Furthermore, the expression of glutamine synthetase and superoxide dismutase were significantly increased after differentiation (*p* < 0.05; Supplementary Tables 2, 3).

**TABLE 1:** Significantly upregulated proteins during the odonto/osteogenic differentiation of SCAPs (fold change ≥ 1.5; *P < 0.05, **P < 0.01).

	Accession	Protein name	Σ Coverage	Σ PSMs	Fold change (SCAP-induce/SCAP)	Fold change (BMSC-induce/BMSC)	Fold change (SCAP/BMSC)
Proteins also significantly upregulated during the differentiation of BMSCs	O94985	Calsyntenin-1	32.42	173	2.0**	1.2**	0.7**
	Q8WVQ1	Soluble calcium-activated nucleotidase 1	41.90	45	1.5**	1.3**	0.9*
	Q9BRF8	Calcineurin-like phosphoesterase domain-containing protein 1	33.44	43	1.6**	2**	1
	Q9H4F8	SPARC-related modular calcium-binding protein 1	13.82	15	1.6**	1.5**	2.7**
	O00299	Chloride intracellular channel protein 1	64.73	64	1.5**	1.3**	1.8**
	P55290	Cadherin-13	25.25	48	1.7**	1.3**	0.7**
	P11717	Cation-independent mannose-6-phosphate receptor	22.04	209	2.1**	1.7**	0.8**
	P01344	Insulin-like growth factor II	61.67	69	1.7**	3.7**	1.4**
	P18065	Insulin-like growth factor-binding protein 2	53.54	170	2.5**	3.7**	0.5**
	O95633	Follistatin-related protein 3	30.04	30	2.4**	1.9**	0.3**
	P26022	Pentraxin-related protein PTX3	51.97	107	2.3**	1.1**	0.4**
	P02795	Metallothionein-2	50.82	25	5.1*	2.0*	1.1
	O43493	Trans-Golgi network integral membrane protein 2	35	56	2.0**	1.5**	0.7**
	O95965	Integrin beta-like protein 1	53.04	124	1.7**	1.3**	0.5**
	P35556	Fibrillin-2	37.05	476	1.8**	2.1**	1.1*
	Q13443	Disintegrin and metalloproteinase domain-containing protein 9	30.53	70	1.8**	1.3**	0.9
	Q9UBP4	Dickkopf-related protein 3	50	183	2.1**	1.4**	1.1
	Q99523	Sortilin	18.77	113	2.8**	2.9**	0.8**
	Q6UVY6	DBH-like monooxygenase protein 1	19.25	43	2.1**	1.1**	0.5**
	P00325	Alcohol dehydrogenase 1B	19.73	37	2.1**	1.8**	1.5**
	Q13740	CD166 antigen	25.21	59	2.4**	1.9**	0.7**
	P05090	Apolipoprotein D	32.8	62	3.1**	3.8**	0.9**
	P04080	Cystatin-B	48.98	45	1.6**	1.3**	1.1
	P16035	Metalloproteinase inhibitor 2	60.45	226	1.7**	1.2**	1.1
	Q01995	Transgelin	97.01	238	1.6**	1.2**	1.1
Proteins significantly downregulated during the differentiation of BMSCs	P07858	Cathepsin B	30.68	106	2**	2.2**	0.8**
	Q86SX6	Glutaredoxin-related protein 5	42.68	19	1.8*	6*	3.2*
	P61812	Transforming growth factor beta-2	30.19	68	2.2**	0.6**	2.1**
	P19438	Tumor necrosis factor receptor superfamily member 1A	18.68	18	2.5*	0.7*	1
	P25205	DNA replication licensing factor MCM3	30.69	52	2.1*	0.8*	1.4*
	P01011	Alpha-1-antichymotrypsin	20.8	23	2.7**	0.8*	0.3**
	Q9HCL0	Protocadherin-18	14.54	92	1.5**	0.7**	1.2
	Q13642	Four and a half LIM domains protein 1	40.25	69	2**	0.6**	2.2**
	Q9NZP8	Complement C1r subcomponent-like protein	22.59	58	1.6**	0.8**	0.5**
Proteins with no change during the differentiation of BMSCs	Q9UN70	Protocadherin gamma-C3	17.88	44	2.3**	1.0	0.9*
	P39059	Collagen alpha-1(XV) chain	15.06	77	2.1**	0.9	1.6**
	Q9H0B8	Cysteine-rich secretory protein LCCL domain-containing 2	24.95	19	3.0*	1.0	0.6*
	P48740	Mannan-binding lectin serine protease 1	28.76	126	2.4**	1.2	0.8**
	P52823	Stanniocalcin-1	35.22	33	1.8**	1	0.5**
	Q9UNW1	Multiple inositol polyphosphate phosphatase 1	30.8	52	2**	1	0.9
	P55058	Phospholipid transfer protein	36.71	103	1.8**	1.2	1.2**
	P35520	Cystathionine beta-synthase	28.49	71	2*	0.9	1.9*

In total, 47 proteins were significantly downregulated (*p* < 0.05) during the differentiation of SCAPs. Some of these are listed in Table 2. The expression of signaling molecules such as IGF-binding protein 3 and TGFβ-induced protein ig-h3 were decreased during the differentiation of SCAPs and BMSCs, as well as tubulin beta-3 chain, midkine, and stromal cell–derived factor 1. Meanwhile, the expressions of periostin and heat shock protein 90 were significantly decreased only during the differentiation of SCAPs.

**TABLE 2:** Significantly downregulated proteins during the odonto/osteogenic differentiation of SCAPs (fold change ≥ 1.5; *P < 0.05, **P < 0.01).

	Accession	Protein name	Σ Coverage	Σ PSMs	Fold-change (SCAP/SCAP-induce)	Fold-change (BMSC/BMSC-induce)	Fold-change (SCAP/BMSC)
Proteins significantly downregulated during the differentiation of BMSCs	Q9H013	Disintegrin and metalloproteinase domain-containing protein 19	17.28	48	2.3*	3.6*	2.25*
	Q15582	Transforming growth factor-beta-induced protein ig-h3	42.75	157	2.2**	2.0**	0.72**
	Q6FHJ7	Secreted frizzled-related protein 4	48.55	87	2.5**	2.6**	0.93
	Q96SM3	Probable carboxypeptidase X1	8.99	23	3.8**	3.3**	1.7**
	Q15436	Protein transport protein Sec23A	22.75	48	2.2**	1.8**	1.07
	P03956	Interstitial collagenase	39.87	53	2.3**	4.6**	0.59**
	P21741	Midkine	62.24	45	2.3**	1.5**	3.67**
	O60814	Histone H2B type 1-K	43.65	24	6.0*	2.3*	4.86*
	Q96CX2	BTB/POZ domain-containing protein KCTD12	61.85	72	2.4**	1.3**	1.98**
	P39687	Acidic leucine-rich nuclear phosphoprotein 32 family member A	30.12	22	2.3*	2.4*	0.72*
	P13667	Protein disulfide-isomerase A4	30.23	63	2.4**	1.4*	0.64**
	P55316	Forkhead box protein G1	28.63	40	2.3*	2.0*	1.35*
	P48061	Stromal cell-derived factor 1	39.78	12	2.2**	1.6**	2.26**
	Q96RW7	Hemicentin-1	17.21	231	2.2**	1.9**	1.09*
	P06576	ATP synthase subunit beta, mitochondrial	15.5	26	1.9*	1.8*	1.13*
	P13611	Versican core protein	15.22	238	1.9**	1.7**	0.89**
	P35442	Thrombospondin-2	37.29	174	1.9**	1.7**	0.46**
	P49006	MARCKS-related protein	20.51	28	2*	1.6*	2.62*
	Q13509	Tubulin beta-3 chain	35.56	58	1.6*	2.4*	1.4*
	P17936	Insulin-like growth factor-binding protein 3	60.14	86	2**	1.5**	0.9*
	Q15847	adipogenesis regulatory factor	36.84	8	1.6*	1.9*	0.6*
	Q9NRR1	cytokine-like protein 1	34.56	20	1.6**	2**	0.4**
Proteins significantly upregulated during the differentiation of BMSCs	P81605	Dermcidin	50	41	3.7**	0.4**	1.06
	P07900	Heat shock protein HSP 90-alpha	32.1	91	2.2**	0.9**	0.78
	P55072	Transitional endoplasmic reticulum ATPase	39.33	94	2**	0.8**	1.34**
Proteins with no change during the differentiation of BMSCs	P27797	Calreticulin	32.61	64	2.5**	0.9	0.61**
	Q15063	Periostin	62.2	397	4.0**	0.9	3.82**
	P36222	Chitinase-3-like protein 1	27.94	35	2.7**	1.2	3.73**
	P08238	Heat shock protein HSP 90-beta	40.33	137	2.1**	1.1	1.76**

When comparing the secretomes of SCAPs and BMSCs, we found significant differences of at least 100% (*p* < 0.05) for 151 proteins. Of these, 83 were higher in SCAPs, and 68 were lower (Yu et al., 2016). When we compared these proteins with the proteins that significantly changed during odonto/osteogenic differentiation, we found that glutaredoxin-related protein 5 (GLRX5) was the only protein secreted more by SCAPs than BMSCs (significant differences of at least 100%) and significantly upregulated during the differentiation of both cell types (significant differences of at least 50%). TGFβ2 and Four and a half LIM domains protein 1 (FHL1) were secreted more by SCAPs than BMSCs, were significantly upregulated during the differentiation of SCAPs and were significantly downregulated during the differentiation of BMSCs. Thrombospondin-2 (THBS2), adipogenesis regulatory factor (ADIRF), and cytokine-like protein 1 (CYTL1) were secreted less by SCAPs than BMSCs and were significantly downregulated during the differentiation of both cell types.

### Bioinformatics Analysis of the Secretome

Among the 92 proteins upregulated during the odonto/osteogenic differentiation of SCAPs, 40 matched the GO term “extracellular region” according to the DAVID Bioinformatics Resources. To facilitate the interpretation of these proteins, a functional interaction network of ECM proteins was searched using the String software. As illustrated in Figure 2B, the ECM proteins secreted by SCAPs belonged to seven major functional classes: ECM organization, negative regulation of cell proliferation, cell adhesion, negative regulation of signal transduction, calcium ion binding, immune response, and enzyme regulator and inhibitor. The interaction network of differentially expressed genes indicated that matrix metalloproteinase2, serpin family E member 1, metallopeptidase inhibitor 2, decorin, cystatin C, TGFβ2, and IGF2 were the most important genes in the network with powerful mediation ability. Among these genes, TGFβ2 was a key node with interaction with six proteins.

In addition, the 47 proteins downregulated during the differentiation of SCAPs matched the GO term “biological process” according to DAVID Bioinformatics Resources and were involved in macromolecular complex assembly, protein localization, and biological adhesion. According to GO analysis in the protein class domain using the PANTHER classification system, the majority of proteins upregulated during both odonto/osteogenic differentiation of SCAPs and BMSCs (*p* < 0.05) mapped to the GO terms “receptor,” “signaling molecule,” “enzyme modulator,” “hydrolase,” and “extracellular matrix protein,” while the downregulated proteins in both processes (*p* < 0.05) mainly mapped to the GO terms “signaling molecule” and “nucleic acid binding.”

### Validation of Differentially Secreted Proteins

**FIGURE 3:**
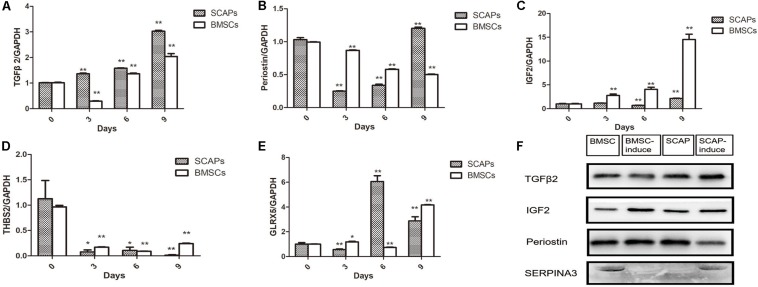
Validation of differentially secreted proteins. **(A–E)** Real-time reverse transcription polymerase chain reaction (RT-PCR) analysis of TGFβ2, periostin, Thrombospondin-2 (THBS2), IGF2, and glutaredoxin-related protein 5 (GLRX5) expression. PCR expression level is represented as the fold change relative to the levels on day 0. All data are presented as the mean ± SD; *p < 0.05, **p < 0.01 versus day 0; each experiment was repeated three times. **(F)** The results of western blotting analysis of TGFβ2, IGF2, periostin, and SERPINA3 were consistent with the findings in our proteomic analysis. TGFβ2 expression was increased after the induction of SCAPs and decreased after the induction of BMSCs at 6 days.

Several of the differentially secreted proteins were validated by real-time RT-PCR and western blotting (Figure 3). TGFβ2 mRNA levels decreased remarkably in the first 3 days after the induction of BMSC differentiation, although this pattern reversed itself, and its expression increased at later time points. In contrast, TGFβ2 mRNA levels increased during the differentiation of SCAPs (Figure 3A). Consistent with findings from our proteomic analysis, western blotting revealed increased secretion of TGFβ2 from SCAPs and decreased secretion from BMSCs, determined at day 6 after the induction of differentiation (Figure 3F). Meanwhile, periostin mRNA levels decreased remarkably in differentiating SCAPs but decreased only slightly in BMSCs, both detected at days 3 and 6 after induction (Figure 3B). At day 6, the secretion of periostin was significantly reduced in SCAPs but did not change significantly in BMSCs (Figure 3F). The results of real-time RT-PCR and western blotting also documented enhanced expression and secretion of IGF2 during the odonto/osteogenic differentiation of both SCAPs and BMSCs (Figures 3C,F). The expression of the THBS2 gene was remarkably decreased in both SCAPs and BMSCs at day 3 after induction of differentiation, and mRNA levels remained low until day 9 (Figure 3D). The mRNA level of GLRX5 also increased remarkably in SCAPs at days 6 and 9 also and also underwent a significant increase in BMSCs, detected at days 3 and 9 (Figure 3E). Finally, secretion of SERPINA3 decreased significantly in BMSCs and increased significantly in SCAPs as demonstrated from findings evaluated at day 6 (Figure 3F).

### Impact of TGFβ1 and TGFβ2 on the Osteo/Odontogenic Differentiation of SCAPs

We performed real-time RT-PCR, western blotting, and alizarin red staining to investigate the effects of exogenous recombinant human TGFβ1 (rhTGFβ1; Peprotech, United States) and recombinant human TGFβ2 (rhTGFβ2; Peprotech, United States) on osteo/odontogenic differentiation of SCAPs and BMSCs. The SCAPs and BMSCs were each divided into four groups: (a) 10% FBS plus aMEM as a control, (b) mineral-inducing medium (OM) alone, (c) OM with 1 ng/ml rhTGFβ2, and (d) OM with 1 ng/ml rhTGFβ1.

**FIGURE 4:**
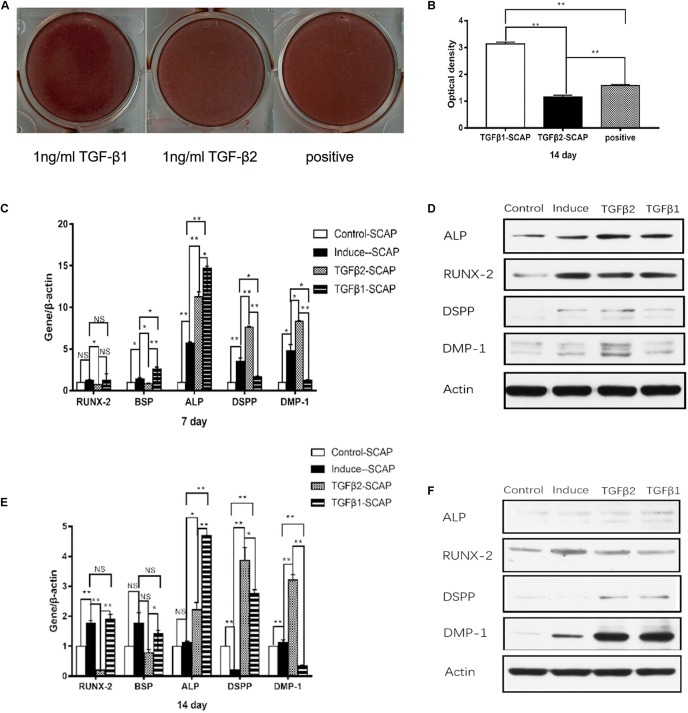
The effects of recombinant human TGFβ1 (rhTGFβ1) and rhTGFβ2 on the osteo/odontogenic differentiation of SCAPs. **(A)** After 2 weeks of culturing in different media, calcium mineral deposition was detected by alizarin red staining. **(B)** Quantitative measurement of alizarin red staining. Mineralization was significantly higher in the TGFβ1 group than in the OM group and significantly lower in the TGFβ2 group than in the OM group. All data are presented as the mean ± SD; **p < 0.01; each experiment was repeated three times. **(C,E)** Real-time RT-PCR showing mRNA level of runt-related transcription factor 2 (RUNX2), ALP, bone sialoprotein (BSP), dentin sialophosphoprotein (DSPP), and dentin matrix protein 1 (DMP1) of SCAPs after culturing in different medium at 7 days **(C)** and 14 days **(E)**. All data are presented as the mean ± SD (*p < 0.05; **p < 0.01; NS, no significance). Each experiment was repeated three times. **(D,F)** Western blotting analysis of expression of RUNX2, ALP, DSPP, and DMP1 of SCAPs after culturing in different medium at 7 days **(D)** and 14 days **(F)**.

After 2 weeks, alizarin red staining and calcium quantitation revealed that SCAP mineralization was significantly higher in the OM with TGFβ1 group and significantly lower in the OM with TGFβ2 group compared to the cells cultured with OM alone (Figures 4A,B). We also examined the expression of ALP, RUNX2, and bone sialoprotein (BSP) in each of these culture conditions (Figures 4C–F). BSP is a key transcription factor involved in osteogenic differentiation. Real-time RT-PCR and western blotting data revealed that both TGFβ2 and TGFβ1 enhanced ALP expression levels. RUNX2 and BSP mRNA levels were significantly lower at days 7 and 14 in the OM with TGFβ2 cell cultures than in those grown in OM alone. In contrast, the BSP mRNA level was significantly higher at day 7 in the OM with TGFβ1 group than in the group cultured in OM alone, although no significant intergroup differences were detected at day 14. RUNX2 mRNA levels detected at days 7 and 14 in SCAPs in OM with TGFβ1 were indistinguishable from those detected in OM culture alone. Taken together, our results suggest that osteogenic differentiation of SCAPs is enhanced by TGFβ1 and attenuated by TGFβ2.

Expression levels of the odontogenesis-specific markers DSPP and DMP1 were also measured by real-time RT-PCR and western blotting (Figures 4C–F). DSPP and DMP1 mRNA and protein levels were significantly higher at days 7 and 14 in the OM with TGFβ2 group compared with the SCAPs cultured in OM alone. DSPP and DMP1 mRNA levels were significantly lower in the OM with TGFβ1 group than in cells cultured in OM alone at day 7, but no differences were identified in their protein levels. At day 14, the SCAPs in OM with TGFβ1 displayed higher DSPP mRNA levels and lower DMP mRNA levels compared with cells cultured in OM alone. Interestingly, higher levels of both DSPP and DMP1 proteins were detected in the OM with TGFβ1 group at this time point. As such, we conclude that TGFβ2 may enhance the odontogenic differentiation of SCAPs, whereas TGFβ1 may attenuate the early odontogenic differentiation of SCAPs while promoting differentiation at a later stage.

### Impact of TGFβ1 and TGFβ2 on the Osteo/Odontogenic Differentiation of BMSCs

**FIGURE 5:**
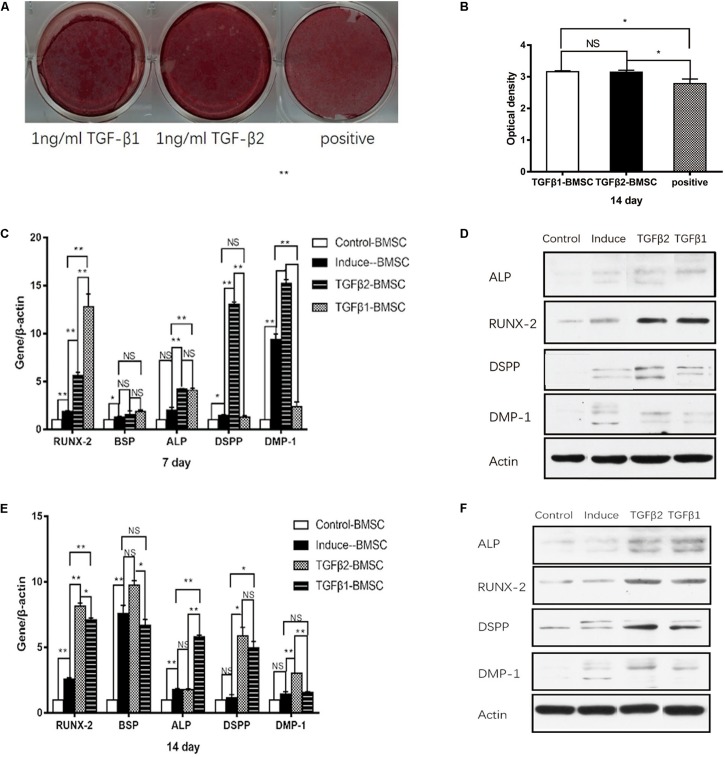
The effects of rhTGFβ1 and rhTGFβ2 on the osteo/odontogenic differentiation of BMSCs. **(A)** After 2 weeks of culturing in different media, calcium mineral deposition was detected by alizarin red staining. **(B)** Quantitative measurement of alizarin red staining. Increased mineralized nodule was observed in the presence of 1 ng/ml rhTGFβ1 and 1 ng/ml rhTGFβ2 compared with OM at 14 days, with no significant difference between TGFβ1 and TGFβ2 groups. All data are presented as the mean ± SD (*p < 0.05; NS, no significance). Each experiment was repeated three times. **(C,E)** Real-time RT-PCR showing mRNA level of RUNX2, ALP, BSP, DSPP, and DMP1 of BMSCs after culturing in different medium at 7 days (Figure 4C) and 14 days (Figure 4E). All data are presented as the mean ± SD (*p < 0.05; **p < 0.01; NS, no significance). Each experiment was repeated three times. **(D,F)** Western blotting analysis of expression of RUNX2, ALP, DSPP, and DMP1 of BMSCs after culturing in different medium at 7 days (Figure 4D) and 14 days (Figure 4F).

The findings shown in Figures 5A,B reveal increased mineralized nodule formation at day 14 in BMSCs cultured in OM with TGFβ1 and in OM with TGFβ2 compared with those cultured in OM alone; no significant differences were detected when comparing the results from OM with TGFβ1 cultures to those from OM with TGFβ2. Real-time RT-PCR and western blotting documented significantly elevated RUNX2 expression at days 7 and 14 in both the OM with TGFβ1 and OM with TGFβ2 groups compared with those from BMSCs maintained in OM alone (Figures 5C–F). In contrast, BSP mRNA levels remained stable and showed no response to TGFβ1 or TGFβ2 (Figures 5C–F). At day 7, ALP mRNA and protein levels were higher in the cultures maintained in OM with TGFβ1 or TGFβ2 than in those maintained in OM alone (Figures 5C,D). At day 14, ALP mRNA and protein levels were significantly higher in the cells cultured in OM with TGFβ1 compared with those cultured in OM alone (Figures 5E,F). Similarly, ALP protein level was higher in the OM with TGFβ2 group than in the OM alone group; however, we detected no significant differences in ALP mRNA levels.

The findings in Figures 5C–E indicate that DSPP and DMP1 protein levels detected at days 7 and 14 were significantly higher in the BSMCs cultured in OM with TGFβ2 than in the group maintained in OM alone. At day 7, no changes in DSPP mRNA levels were detected when comparing results from OM with TGFβ1 to those cultured in OM alone. However, at day 14, it was significantly higher in the TGFβ1 than in the OM group. DMP1 mRNA and protein levels were significantly lower in the TGFβ1 with OM cultures than in those maintained in OM alone at day 7, whereas no significant differences were detected at day 14. From these findings, we conclude that TGFβ2 had a greater odontogenic impact on BMSCs compared with TGFβ1.

## Discussion

Cells with characteristics of multipotent progenitors have been identified in stromal tissues. These progenitors are described as MSCs if they are capable of self-renewal and generation of multiple lineages (Gronthos et al., 2000; Bianco et al., 2001; Crisan et al., 2008; Sonoyama et al., 2008). When grown under mineralizing conditions, MSCs are capable of secreting a mineralized matrix that is used as an indication of their potential for “osteogenic” differentiation. Previous studies have confirmed that MSCs derived from individual tissues have distinct features (Gronthos et al., 2000; Seo et al., 2004; Huang et al., 2010). SCAPs are regarded as the source of primary odontoblasts and promote regeneration of the dentin matrix *in vivo* (Huang et al., 2008; Sonoyama et al., 2008; Huang et al., 2010). The evaluation of odontogenic and osteogenic differentiation of MSCs is commonly carried out by an illustration of the expression of varied related markers. When grown under mineralizing conditions *in vitro*, SCAPs undergo osteo/odontogenic differentiation; the expressions of osteoblastic markers and odontoblastic markers are upregulated (Ma et al., 2016; Wang et al., 2018; Zhang et al., 2019). To the best of our knowledge, there are no published systemic proteomic analyses that explore the extracellular environment of SCAPs during osteo/odontogenic differentiation.

Most of the proteins that exhibited significant changes during the osteo/odontogenic differentiation of SCAPs also changed during the differentiation of BMSCs. ECM organization proteins, calcium-binding proteins, and proteins negatively regulating cell proliferation increased significantly during differentiation of both SCAPs and MBSCs, while macromolecular complex assembly proteins and growth factors significantly decreased. In this study, BMSCs were isolated from alveolar bone marrow. Tooth and craniofacial bone arise from neural crest cells. The expressions of neural tissue–related proteins such as tubulin beta-3, midkine, and forkhead box protein G1 all decreased after the induction of differentiation; these findings confirm the differentiation of these MSCs.

As SCAPs are the source of odontoblasts and BMSCs give rise to osteoblasts, we focused our study on the genes and proteins that were differentially expressed during the differentiation; our intent was to reveal distinct regulatory mechanisms responsible for odontogenesis and osteogenesis. In the present study, GLRX5 was upregulated during the differentiation of both SCAPs and BMSCs but was secreted in higher concentrations by the SCAPs. GLRX5 is a mitochondrial protein that contributes to the generation of iron–sulfur complex and plays a multifunctional role in iron–sulfur protein synthesis and maturation (Liu et al., 2016); differential secretion of GLRX5 may be associated with varying capacities for supporting biosynthesis of iron/sulfur complexes.

THBS2, ADIRF, and CYTL1 were secreted by SCAPs albeit to a lesser extent than observed in BMSCs; these factors were downregulated during the differentiation of SCAPs and BMSCs. THBS2 is an autocrine inhibitor of MSC proliferation. THBS2-null cells demonstrate an increased rate of proliferation, whereas addition of recombinant THBS2 resulted in a dose-dependent decrease in MSC proliferation (Hankenson and Bornstein, 2002), although the rate of proliferation of SCAPs was higher than that of BMSCs (Yu et al., 2016). When MSCs undergo differentiation, their proliferation is inhibited. ADIRF (also known as APM2 or C10orf116) is also highly expressed in adipose tissue, where it promotes early-stage adipogenic differentiation (Ni et al., 2013). Likewise, CYTL1, a factor originally identified in CD34^+^ bone marrow and cord blood mononuclear cells, was implicated in promoting neoplastic cell growth and metastasis (Liu et al., 2000; Ai et al., 2016; Wang et al., 2016). Recently, it has been found that CYTL1 negatively regulates the osteogenesis of human MSCs; specific inhibition was demonstrated in response to overexpression or exogenous treatment with CYTL1, and proliferation was enhanced by CYTL1 knockdown (Shin et al., 2019).

We found that FHL1 and TGFβ2 were secreted by SCAPs and, to a lesser extent, by BMSCs; both proteins were upregulated during the differentiation of SCAPs and downregulated during the differentiation of BMSCs. It is interesting to consider the possibility that FHL1 may promote osteogenesis; siRNA knockdown of FHL1 resulted in a reduction in mineralized deposits and downregulated the expressions of RUNX2, OPN, and OCN at day 14 (Wu et al., 2015; Hemming et al., 2016). The gene encoding FHL1 contains TCF/LEF transcription factor binding sites and is a downstream target of Wnt signaling in differentiating muscle cells (Lee et al., 2012). However, others have shown that the canonical Wnt signaling pathway is a crucial regulator of osteoprogenitor proliferation and suppresses final osteoblast maturation (Boland et al., 2004; De Boer et al., 2004). In addition, FHL1 regulates calcium homeostasis (Pillar et al., 2017). We suggest that FHL1 plays a complex, multifaceted role in both odontogenesis and osteogenesis.

According to GO analysis, TGFβ2 is one of the most important proteins in the interaction network of ECM proteins. TGFβs are secreted proteins that are recognized as key regulators of stem cell renewal and differentiation (Kumar and Sun, 2005; Mishra et al., 2005; Zhou, 2011). Three mammalian isoforms (TGFβ1, β2, and β3) have been identified in bone and dentin. TGFβ1 predominates among the TGFβ isoforms that have been identified in dentin and bone (Seyedin et al., 1985; Cassidy et al., 1997). TGFβ1 is expressed in dental epithelia and in the mesenchyme at the bud and cap stages and has also been detected in developing teeth from the initiation stage through adulthood (Benedete et al., 2008; Huang and Chai, 2010; Li and Pan, 2017). TGFβ2 expression has been detected in the dental papilla and preodontoblasts but not in the dental epithelium (Benedete et al., 2008; Huang and Chai, 2010). In the tooth germ of a 6-month-old permanent incisor in a porcine species, TGFβ1 mRNA was predominantly detected in odontoblasts, whereas TGFβ2 mRNA was identified at high levels in the dental pulp tip (Niwa et al., 2018).

In mice overexpressing TGFβ1 in teeth, significant reduction in tooth mineralization, defective dentin formation, increased levels of calcium, and significantly diminished expression of DSPP have been reported (Thyagarajan et al., 2001). A previous study of TGFβ2-overexpressing mice documented increased dentin formation and bone mineral apposition rate, without any significant change in dentin microhardness and a more porous, osteoporotic phenotype in the bone (Denbesten et al., 2001). Likewise, in TGFβ receptor 2 gene–deleted mice, a substantial delay in odontoblast differentiation, decreased dentin thickness, and absent dentinal tubules in tooth germs were reported, whereas exogenous TGFβ2 induced nestin and DSPP expression in dental pulp cells (Oka et al., 2007). From these studies, we hypothesize that the roles of TGFβ1 and TGFβ2 in promoting odontogenesis differ markedly from one another.

BSP is a major ECM component of the bone. BSP is produced mainly by osteoblasts and is considered to be a marker for osteoblastic phenotypes (Mizuno et al., 2000). In contrast, DSPP is differentially expressed in the dentin tissue. In this study, we found that administration of TGFβ2 resulted in enhanced expression of the odontogenic-related markers DSPP and DMP1 in both SCAPs and BMSCs, and downregulated the expression of osteogenic-related markers BSP in SCAPs. It is speculated that TGFβ2 can promote the odontogenic differentiation of MSCs. Meanwhile, TGFβ1 enhanced the BSP expression and inhibited DSPP and DMP1 expression during early odonto/osteogenic differentiation of SCAPs but upregulated the expression of DSPP in SCAPs at the later stage. The BSP and DSPP expression levels did not differ between the OM with TGFβ1 group and OM alone group at the early time points of odonto/osteogenic differentiation of BMSCs, and TGFβ1 upregulated the expression of DSPP in BMSCs at the later stage. Hence, TGFβ1 may play a positive role in the mature stage of odontogenic differentiation of MSCs.

In human bone samples, levels of TGFβ1 protein ranged between 27 and 580 ng/g bone and for TGFβ2 between 7.2 and 35 ng/g bone (Hering et al., 2001). In contrast to TGFβ1, TGFβ2 mRNA expression was significantly enhanced in osteoarthritic bone compared to unaffected bone (Hering et al., 2001). TGFβ2 reduces bone and cartilage formation in healing fractures in rabbits (Critchlow et al., 1995). Previous research showed that the osteogenic differentiation of inflamed dental follicle stem cells (DFSCs) was suppressed, and the cells displayed low levels of TGFβ1 and high levels of TGFβ2 (Um et al., 2018). DFSCs treated with TGFβ2 inhibitors showed significant increases in alizarin red staining and ALP activity (Um et al., 2018). TGFβ1 expression was also increased after inhibition of TGFβ2 (Um et al., 2018). It is speculated that TGFβ2 may inhibit bone formation.

Although TGFβ1 and TGFβ2 are highly homologous at the level of sequence, analysis of their *in vivo* function by gene knockouts revealed striking differences, suggesting no significant functional redundancy between TGFβ1 and TGFβ2 (Sanford et al., 1997; Denbesten et al., 2001; Thyagarajan et al., 2001). Previous research demonstrated that TGFβ2 operates through a distinct mechanism in which the type III receptor functions as a co-receptor for efficient binding, in contrast to TGFβ1/3, which acts by direct binding to type I and II receptors (Zuniga et al., 2005). It is reported that TβRII-B, an alternatively spliced variant of the TGFβ type II receptor, is a TGFβ2 binding receptor, which mediates signaling via the Smad pathway in the absence of any TGFβ type III receptor (Rotzer et al., 2001). The expression of TβRII-B is restricted to cells originating from tissues such as bone where TGF2 has a predominant role (Rotzer et al., 2001).

## Conclusion

In conclusion, we evaluated the secretomes of differentiating SCAPs and BMSCs to understand the molecular events underlying the early steps in dentin matrix formation and to elucidate distinct mechanisms in early odontogenesis and osteogenesis. Our findings identified a set of proteins that are differentially secreted during early odontogenesis and osteogenesis and suggest that the differentiation of odontoblasts and osteoblasts and secretion of a mineralized matrix are controlled by complex regulatory mechanisms. Among our findings, TGFβ2 enhanced the odontogenic differentiation and attenuated the osteogenic differentiation of SCAPs, while capable of enhancing odontogenic but not osteogenic differentiation of BMSCs. Taken together, our results suggest that TGFβ2 may induce pulp regeneration to promote dentin regeneration. Overall, we have identified TGFβ2 as a potentially important molecule with distinct functions promoting the regulation of MSC differentiation.

## Data Availability Statement

All datasets generated for this study are included in the article/Supplementary Material.

## Ethics Statement

The studies involving human participants were reviewed and approved by the Ethics Committee of the Health Science Center of Peking University (Beijing, China; IRB00001052-11060 and PKUSSIRB-201734036). Written informed consent to participate in this study was provided by the participants’ legal guardian/next of kin.

## Author Contributions

SY contributed the conception and design of the study. SY and JL organized the database, performed the statistical analysis, and wrote the manuscript. All authors contributed to manuscript revision, read, and approved the submitted version.

## Conflict of Interest

The authors declare that the research was conducted in the absence of any commercial or financial relationships that could be construed as a potential conflict of interest.

## Footnotes

1 http://string-db.org/

2 http://david.abcc.ncifcrf.gov/

3 www.pantherdb.org/
