# Supplementary material for: Comparative Secretome Analysis of Mesenchymal Stem Cells From Dental Apical Papilla and Bone Marrow During Early Odonto/Osteogenic Differentiation: Potential Role of Transforming Growth Factor-β2
Source: Front Physiol. 2020 Mar 6;11:41. doi: 10.3389/fphys.2020.00041 (PMC7073820; doi:10.3389/fphys.2020.00041)
Supplement: Supplementary file 1 [file Table_1.DOCX]

| Gene Symbol | Primer Sequences (5'--3') |
| --- | --- |
| OCN-F | CACTCCTCGCCCTATTGGC |
| OCN -R | CCCTCCTGCTTGGACACAAAG |
| RUNX2-F | TGGTTACTGTCATGGCGGGTA |
| RUNX2-R | TCTCAGATCGTTGAACCTTGCTA |
| ALP-F | ATGGGATGGGTGTCTCCACA |
| ALP -R | CCACGAAGGGGAACTTGTC |
| BSP-F | CAGGCCACGATATTATCTTTACA |
| BSP-R | CTCCTCTTCTTCCTCCTCCTC |
| DSPP-F | CGACATAGGTCACAATGAGGATGTCG |
| DSPP-R | TTGCTTCCAGCTACTTGAGGTC |
| DMP1-F | CGTGGACAAAGAAGATAGCAACTCCACG |
| DMP1-R | TTCCGGCTCTCTATCTCAATGTTT |
| IGF2-F | TTCAACAAGCCCACAGGGTA |
| IGF2-R | GCAATACATCTCCAGCCTCCT |
| THBS2-F | GACACGCTGGATCTCACCTAC |
| THBS2-R | GAAGCTGTCTATGAGGTCGCA |
| GLRX5-F | AAGGACAAGGTGGTGGTCTCT |
| GLRX5-R | TCAGTTCTTCCACCAAGTCCC |
| Periostin-F | CTCATAGTCGTATCAGGGGTCG |
| Preiostin-R | ACACAGTCGTTTTCTGTCCAC |
| TGFβ2-F | CAGCACACTCGATATGGACCA |
| TGFβ2-R | CCTCGGGCTCAGGATAGTCT |
| GAPDH-F | CGAACCTCTCTGCTCCTCCTGTTCG |
| GAPDH-R | CATGGTGTCTGAGCGATGTGG |
| β-actin-F | CCTGGCACCCAGCACAAT |
| β-actin-R | GGGCCGGACTCGTCATACT |

Supplementary Table 1. Primer sequences used for real-time RT-PCR.
